# Chromate Reduction in Serratia marcescens Isolated from Tannery Effluent and Potential Application for Bioremediation of Chromate Pollution

**DOI:** 10.1100/tsw.2002.154

**Published:** 2002-04-09

**Authors:** M.A. Mondaca, V. Campos, R. Moraga, C.A. Zaror

**Affiliations:** Microbiology Department, University of Concepción, P.O. Box 152-C, Correo 3, Concepción, Chile; Chemical Engineering Department, University of Concepción, P.O. Box 152-C, Correo 3, Concepción, Chile

**Keywords:** chromate pollution, chromate reduction, biofilm

## Abstract

Pollution of aquatic systems by heavy metals has resulted in increasing environmental concern because they cannot be biodegraded. One metal that gives reason for concern due to its toxicity is chromium. Cr(VI) and Cr(III) are the principal forms of chromium found in natural waters. A chromate-resistant strain of the bacterium S. marcescens was isolated from tannery effluent. The strain was able to reduce Cr(VI) to Cr(III), and about 80% of chromate was removed from the medium. The reduction seems to occur on the cell surface. Transmission electron microscopic examination of cells revealed that particles were deposited on the outside of bacterial cells. A stable biofilm was formed in less than 10 h, reaching around 10^10^ cfu attached per milligram of activated carbon. These findings demonstrate that immobilized *S. marcescens* might be used in industrial waste treatment processes.

## INTRODUCTION

Widespread pollution by heavy metals has important consequences for human health and environmental quality. Manufacturing processes and domestic wastewater are major sources of chromium contamination[1]. Cr(VI) and Cr(III) are the principal forms of chromium found in natural waters. Cr(VI) is a highly soluble and toxic agent that is also mutagenic and carcinogenic, whereas Cr(III) is much less toxic and tends to form insoluble hydroxides[2]. Therefore, reduction of Cr(VI) to Cr(III) is a potentially useful process for the remediation of chromium-contaminated waters and waste streams. A wide variety of microorganisms can enzymatically reduce Cr(VI) to Cr(III)[3].

Chromate-resistant *Serratia marcescens* bacteria, isolated from a tannery effluent, has been shown to present chromate-reduction activity and can grow in the presence of a wide range of industrial contaminants. The reduction of chromate is a potentially useful process for the remediation of chromium-contaminated waters. The method most often applied to metal removal is the use of cells immobilized as a biofilm on inert supports[4]. The formation of biofilms can be considered as a development cycle. It begins when free-swimming (planktonic) bacteria recognize a surface and firmly attach themselves to it. Subsequently, the attached cells grow and divide, and at the same time recruit additional planktonic cells that attach to the cells already on the surface[5]. Bacterial adhesion is not only of importance in microbial ecology, but also in biotechnology, biofouling, and wastewater treatment. The ability of a bacterium to interact with a given surface is known to be governed by particular molecules or structures on the outer surface of the cell. These may be proteins or lipopolysaccharides in the outer membrane, proteinaceous appendages such as fimbriae or flagella, or a complex carbohydrate in the form of extracellular capsules[6]. Understanding the fundamental processes contributing to biofilm formation is beneficial to anyone involved with natural or industrial systems, where biofilms may play a significant role in determining variables such as bulk water quality, toxic compound biodegradation, or product quality.

The objective of this work was to assess the chromate-reductase activity of S. marcescens isolated from tannery effluent and its ability to attach to an inert surface.

## METHODOLOGY

### Bacteria Strain

A chromate-resistant bacterial strain was isolated from a tannery effluent (Concepción, Chile). The strain was identified as *S. marcescens* using a rapid identification kit (EM-Ident E, SensIdent, Merck Darmstdat, Germany). An overnight culture was diluted in nutrient broth or KSN medium with various concentrations of chromate, and incubation proceeded at 30ºC for 24 h under aerobic conditions. Growth was assayed by measuring turbidity with a spectrophotometer at 660 nm[7].

### Assay of Chromate-Reduction Activity

Samples were withdrawn at different times and centrifuged at 3,000 rpm for 5 min prior to analysis. Cr(VI) and Cr(III) were determined by high pressure liquid chromatography and on-line flame absorption atomic spectroscopy (HPLC-FAAS)[8].

### Hydrophobicity Test

Bacterial adherence was tested using the methods described by Rosenberg et al.[9]. Bacteria suspended in an aqueous phase were mixed with toluene and spun into a vortex. The optical density of the aqueous phase was measured before and after mixing. Results were expressed as the percentage of bacteria excluded from the aqueous phase.

### Attachment of Strain to Activated Carbon (Biofilm Formation)

Cells were cultured until an exponential phase was achieved. Activated carbon, washed with sterile distilled water, was added to the nutrient broth in proportion 5:100 (w/v) and then inoculated with a small aliquot of bacterial inoculum in order to obtain approximately $10^5$ CFU/ml. Incubation was performed at room temperature with constant low-speed shaking. After 3, 24, and 72 h of incubation, duplicated activated carbon samples were washed with distilled water.

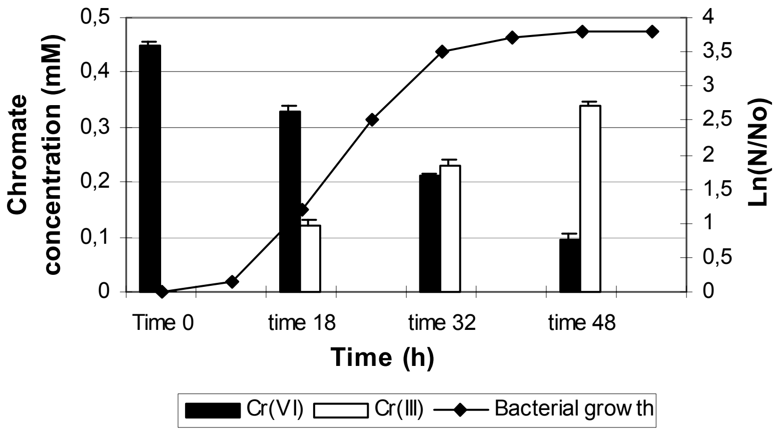

**FIGURE 1.** Reduction of chromate by *S. marcescens* cell culture.

## Scanning Electron Microscopy (SEM)

Samples of activated carbon were fixed with 2.5% glutaraldehyde. Samples were critical-point dried with $CO_2$ and coated with gold (S150 sputter coater). These were observed with an SEM (ETEC Company, autoscan) operating at an accelerating voltage of 20 kV.

## RESULTS AND DISCUSSION

Fig. 1 shows the results of *S. marcescens* cells cultured aerobically in KSC medium containing 0.5 mM chromate. As the cell concentration increases, chromate concentration decreases. *S. marcescens* was able to reduce Cr(VI); about 80% of Cr(VI) was reduced to Cr(III) within 48 h. Cell-free KSC media did not show any reduction of chromate.

Several methods have been proposed to determine hydrophobicity. In the study, we used the BATH test, in which the migration of the bacteria from the aqueous phase depended upon their ability to adhere to the organic phase. The strain gave higher hydrophobicity values (67%), and hydrophobic cells adhered in a greater extent than hydrophilic cells.

A solution containing 105 CFU/ml of exponential phase S. marcescens was cultured in the presence of activated carbon (Filtrasorb 400). The colonization properties of S. marcescens were quantified on the surfaces of activated carbon. As shown in Fig. 2, this strain established a stable biofilm containing about 109 (UFC/g active carbon) in 24 h.

SEM showed a stable biofilm on the carbon surface after 3 h culture and revealed the presence of copious numbers of fibrillar structures, closely associated with the cells, looking like the particular fimbriae described by the Olsen group and designated as curly[10]. Each bacterium was anchored to the carbon surface by means of fibrils in order to bridge the space between the two bodies and allow attachment of the bacterium (Figs. 3A, 3B, and 3C). After 72 h incubation, the attached bacteria excreted extracellular polysaccharides that served as the matrix for the biofilms and covering sessile cells[11]. Similar results were obtained when using water-insoluble polymers as support for a biofilm of *S. marcescens* (Figs. 4A, 4B, and 4C).

Fig. 5 shows chromate reduction by immobilized *S. marcescens* on activated carbon. About 86% of chromate was removed from the medium.

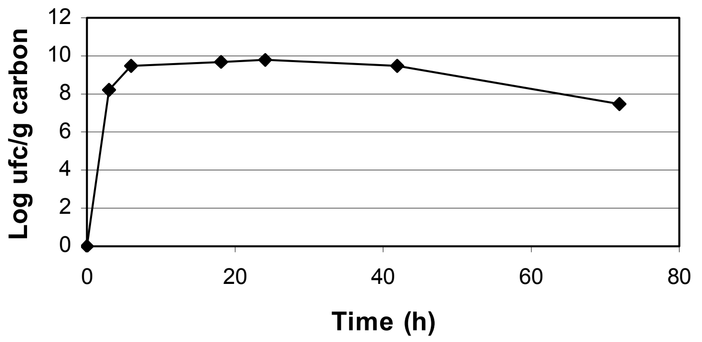

**FIGURE 2.** Growth of *S. Marcescens* attached on activated carbon.

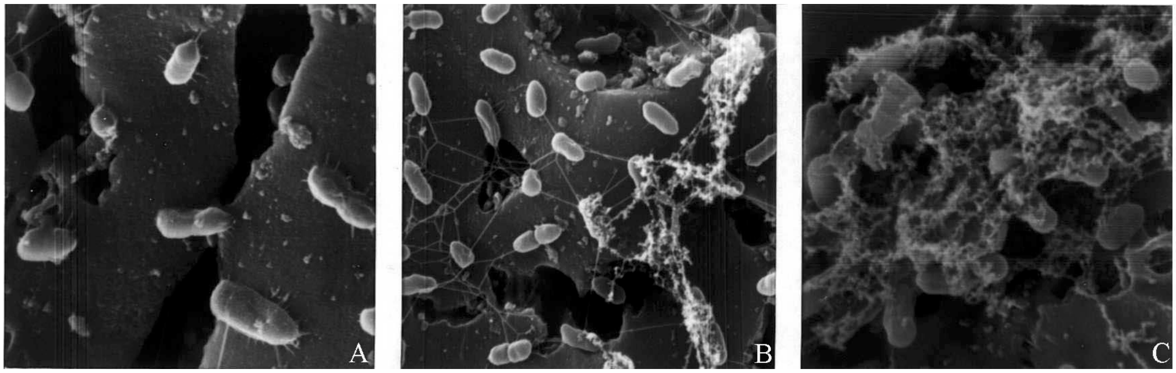

**FIGURE 3.** Biofilm formation of *S. marcescens* on activated carbon. (a) 3 h (b) 24 (c) 72 h.

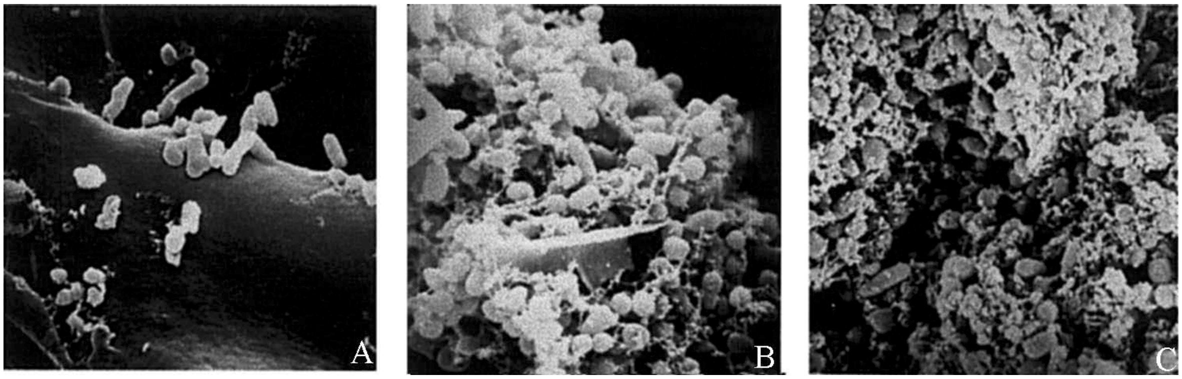

**FIGURE 4.** Biofilm formation of *S. marcescens* on synthetic polymer. (a) 24 h (b) 72 h (c) 96 h.

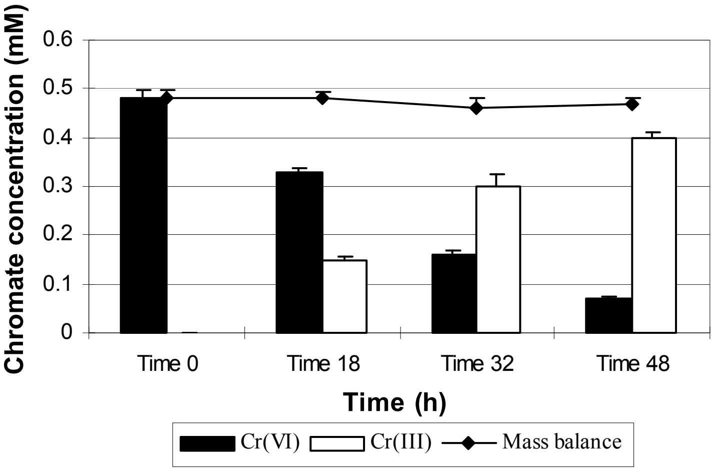

**FIGURE 5.** Chromium (VI) reduction by S. marcescens immobilized on activated carbon with initial 0.5 mM potassium chromate at 30ºC.

## CONCLUSIONS

*S. marcescens* is able to reduce Cr(VI) to Cr(III). It firmly attaches to activated carbon particles and forms a stable biofilm. These findings demonstrate the possibility that immobilized *S. marcescens* might be used in bioremediation of Cr(VI)-contaminated industrial effluent.

## ACKNOWLEDGMENTS

This research was fully supported by the Dirección de Investigación, Universidad de Concepción (Project No. 99.036.016-1.0).
